# PRACTICE CHANGE OUTCOMES OF POST-GRADUATE TRAINING IN ADVANCED CLINICAL DEMENTIA PRACTICES

**DOI:** 10.1093/geroni/igac059.3073

**Published:** 2022-12-20

**Authors:** Daniel Kaplan, Keith Chan

**Affiliations:** Adelphi University, Cortlandt Manor, New York, United States; Hunter College, City University of New York, New York City, New York, United States

## Abstract

Neurocognitive disorder is common among older adults with often progressive symptoms impacting multiple domains of life and with major implications and challenges for family involved in care. Clinical providers including doctors, nurses, and social workers play instrumental roles in addressing health and mental health vulnerabilities associated with neurocognitive disorder as skillful interventions can effectively improve wellness and quality of life for patients and care partners. Yet, providers report needing expanded knowledge to deliver appropriate clinical dementia care. An advanced clinical dementia practice certificate program at a major university in the U.S. offers a 45-hour training that covers 35 clinical practices identified by an interprofessional advisory group. This study aims to test the learning impacts of the educational program. Since 2017, 74 participants have completed this training. Their self-reported frequency of use of each clinical practice was measured before and after the training using a 5-item rating scale ranging from “Never” to “Several times per day”. Comparisons across two time periods demonstrate significant increases in overall use of clinical practices (mean difference: 0.45), with a large mean effect size (Cohen’s d = 0.8). These increases were statistically significant in 30 of 35 practices (p < 0.05). The largest effects were observed for clinical practices emphasizing the teaching of practical care strategies and the application of person-centered interventions. Implications of this educational method for enhancing clinician practice behaviors and recommendations for quantitative evaluation of training outcomes will be presented.

